# Supplementary material for: Folate-Functionalization Enhances Cytotoxicity of Multivalent DNA Nanocages on Triple-Negative Breast Cancer Cells
Source: Pharmaceutics. 2022 Nov 26;14(12):2610. doi: 10.3390/pharmaceutics14122610 (PMC9786333; doi:10.3390/pharmaceutics14122610)
Supplement: Supplementary file 1 [file pharmaceutics-14-02610-s001.zip › pharmaceutics-2015002-supplementary.pdf]

Supplementary Materials

Folate-functionalization enhances cytotoxicity of multivalent DNA nanocages on triple negative breast cancer cells

Valeria Unida[1], Giulia Vindigni[1], Sofia Raniolo[1#], Carmine Stolfi[1], Alessandro Desideri[2] and Silvia Biocca[1*]

[1]Department of Systems Medicine, University of Rome Tor Vergata, Via Montpellier 1, 00133, Rome, Italy. [2]Department of Biology, University of Rome Tor Vergata, Via della Ricerca Scientifica 1, 00133, Rome, Italy.

[*] Correspondence should be addressed to S.B.: biocca@med.uniroma2.it; Dpt. Systems Medicine, University of Rome Tor Vergata, Via Montpellier 1, 00133, Rome, Italy; Tel +39 06 72596418; Fax: +39 06 72596407

[#] Current address: Department of Engineering, Università Campus Bio-Medico di Roma, Via Alvaro del Portillo, 21 - 00128 Roma, Italy

S1. Sequences of oligonucleotides for DNA nanostructures assembly

All oligonucleotides reported in Table S1 were HPLC purified and purchased from LGC Biosearch Technologies (Risskov, DK). The 5' of each oligonucleotide is phosphorylated. TTTTT represents a short non-pairing spacer inserted within the strands as a DNA junction at each vertex of the assembled 3D structure. The oligonucleotides OL1, OL2, OL3, OL4 harbour sequestering units complementary to mature miR-21-5p (OL_{miR21}), or a 21-nucleotide scramble sequence (OL_{scr}). The OL5 oligo has been functionalized with the AS1411 aptamer sequences ($OL5_{APT}$), while the OL8 oligo has been functionalized with folic acid through bio-orthogonal conjugation (click) chemistry ($OL8_{FOL}$), provided by Baseclick (Neuried, Germany). The oligonucleotide $OL6_{BIO}$ has a biotin tetra-ethylene-glycol molecule (BtndT). In blue are shown the two T in OL8 used for folate conjugation and in red is depicted the biotinylated T in $OL6_{BIO}$.

Table S1

Oligo	Sequences (5'-3')
$OL1_{miR21}$	GCCACCAGGTTTTTCGATGTCTAAGCTGACCGTCTTTCCTTTTTCAACATCAGTCTGATAAGCTATTTCCTTTTCTGGACCGTGATTCCATGACTTTTTCTTAGAGTT
$OL2_{miR21}$	TGGCTACAGTCTTTCCTTTTTCAACATCAGTCTGATAAGCTATTTCCTTTTCTCGGTCAGCTTAGACATCGTTTTTGAATCCTATGCTCGGACGTTTTTGGCTCACAT
$OL3_{miR21}$	TCACGGTCCTCTTTCCTTTTTCAACATCAGTCTGATAAGCTATTTCCTTTTCTCTATCCGATCGAGGCATGTTTTTCATACTGAGAGCGTTCCGTTTTTGTCATGGAA
$OL4_{miR21}$	CAGATACGCTTTTTCATGCCTCGATCGGATAGTCTTTCCTTTTTCAACATCAGTCTGATAAGCTATTTCCTTTTCTCTGTAGCCAATGTGAGCCTTTTTGTCGCAGTT
$OL1_{scr}$	GCCACCAGGTTTTTCGATGTCTAAGCTGACCGTCGAATATTTCCCCCCCCCCAGAAACCTTCCTGGACCGTGATTCCATGACTTTTTCTTAGAGTT
$OL2_{scr}$	TGGCTACAGTCGAATATTTCCCCCCCCCCAGAAACCTTCCTCGGTCAGCTTAGACATCGTTTTTGAACTCTATGCTCGGACGTTTTTGGCTCACAT
$OL3_{scr}$	TCACGGTCCAATATTTCCCCCCCCCCAGAAACCTTCCTCTATCCGATCGAGGCATGTTTTTCATACTGAGAGCGTTCCGTTTTTGTCATGGAA
$OL4_{scr}$	CAGATACGCTTTTTCATGCCTCGATCGGATAGTCGAATATTTCCCCCCCCCCAGAAACCTTCCTCTGTAGCCAATGTGAGCCTTTTTGTCGCAGTT
OL5	CTCAGTATGTTTTTCGGTTACGGTACAATGCCTTTTTGCCAAGACGTTAGTGTCCTTTTTCGGAACGCT
$OL5_{APT}$	CTCAGTATGTTTTTCGGTTACGGTACAATGCCTTTTTGCCAAGACGTTAGTGTCCTTTTTCGGAACGCTTTTTTTTGGTGGTGGTGGTTGTGGTGGTGGTGG
$OL6_{BIO}$	GGTGTATCGTTTTTGGCATTG**T**ACCGTAACCGTTTTTGCGTATCTGAACTGCGACTTTTTCCACCGAAT
OL7	CGTCTTGCGTTTTTGTATGACGCAGCACTTGCTTTTTCCTGGTGGCAACTCTAAGTTTTTGGACACTAA
OL8	ATAGGATTCTTTTTGCAAGTGCTGCGTCATACTTTTTCGATACACCATTCGGTGGTTTTTCGTCCGAGC
$OL8_{FOL}$	ATAGGATTCTTTTTGCAAGTGC**T**GCGTCATACTT**T**TTCGATACACCATTCGGTGGTTTTTCGTCCGAGC'
AS1411	GGTGGTGGTGGTTGTGGTGGTGGTGG

S2. *In vitro* anti-miRNA opening reaction

Representative DNA gel analysis of NC-*anti*miR21 and NC-*anti*miR155, as an example of another miRNA, incubated for 30 minutes at 37°C with 10 to 40 times molar excess concentration of oligonucleotides complementary to sequences of miR21 and miR155. After incubation, NCs were analysed by DNA gel and visualized. At 10x oligo molar excess, NCs undergo a complete structural conformational change, as evidenced by a slower electrophoretic mobility.

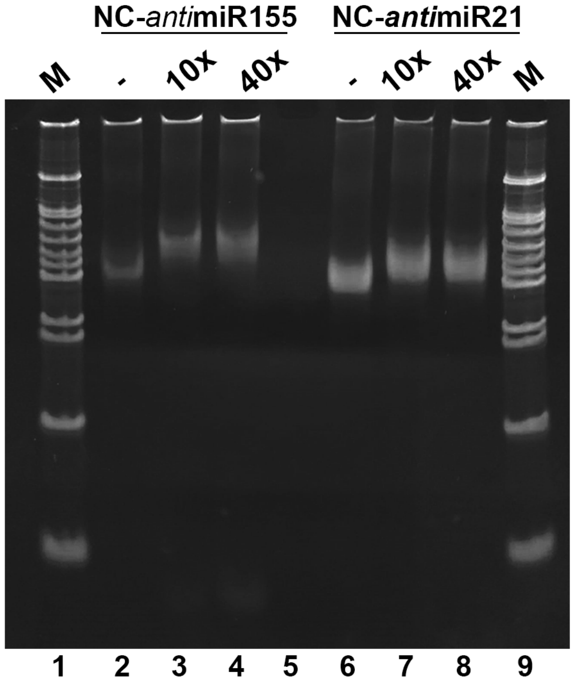

Figure S1 Lanes 2 and 6 show the electrophoretic mobility of 40 ng of closed NC-*anti*miR155 and NC-*anti*miR21, respectively, in the absence of complementary oligonucleotides. Lanes 3, 4 and 7, 8 show the variation of the electrophoretic mobility of the NCs in the presence of increasing concentration, as indicated, of oligonucleotides with miR-155 and miR-21 sequences.

S3. Dose-dependent cytotoxic effect of NC, Fol-NC, Apt-NC, and Fol-Apt-NC on MDA-MB-231 cells

MDA-MB-231 cells were treated for 24 h with increasing amounts of nanocages ranging from 0 to 60 nM. MTS assay shows that NC and Fol-NC are not toxic at these concentrations, while Apt-NC and Fol-Apt-NC display a cell viability reduction which reaches 15.8±3.3% and 20.3±2 respectively, at 60 nM.

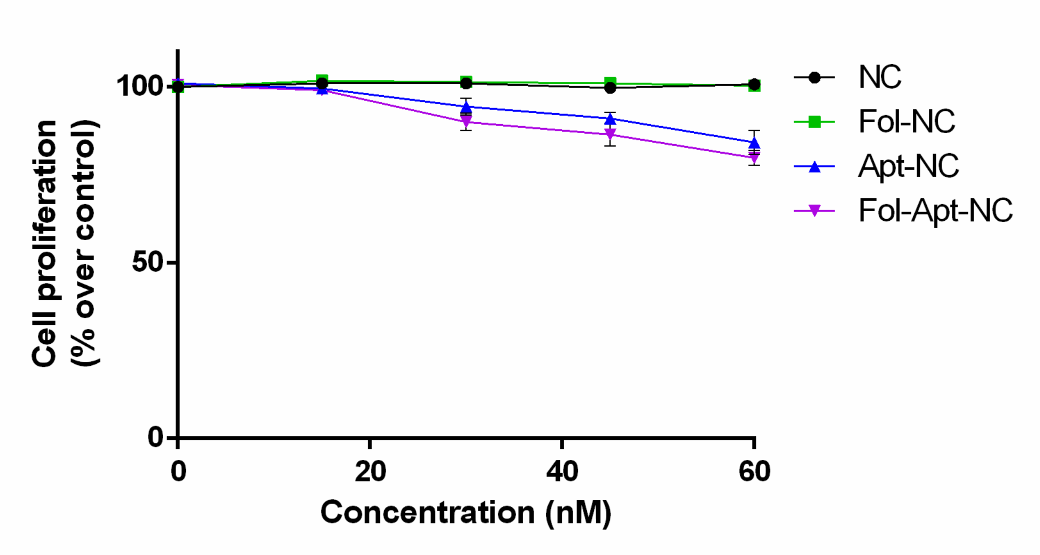

Figure S2 MTS assay of MDA-MB-231 cells treated with increasing concentration of different types of nanocages for 24 h. The data represent mean ± S.E.M. of three separate experiments. The values are the mean of six replicates, normalized on cell proliferation of untreated cells.

S4. Downstream effect of miR21 knockdown in MDA-MB-231

MDA-MB-231 cells were incubated for 48h with 15 nM of either Fol-NC and Fol-NC-*anti*miR21 (Figure S4A) or Apt-NC and Apt-NC-*anti*miR21 (Figure S4B). Figure shows the Western blot of extracts derived from treated cells, to study the expression level of PTEN, a gene regulated by miR21. PTEN rabbit mAb (Cell Signaling Technologies, Danvers, MA, USA, cat. n. 138G6) and β-actin mouse mAb (Cell Signaling Technologies, Danvers, MA, USA, cat. n. 8H10D10) were used as primary antibodies and HRP-conjugated AffiniPure goat anti-mouse IgG and donkey anti-rabbit IgG (Jackson Immunoresearch, Cambridgeshire, UK, cat. n. 115-035-062, cat. n. 711-035-152) were used as secondary antibodies. Protein signals were visualized by enhanced chemiluminescence (ECL Extend, Euroclone, Devon, UK), digitalized and analyzed by ImageJ software. The target protein signal intensities were normalized to β-actin as loading control.

Densitometric analysis (lower panels) indicates that PTEN level is about 2-fold and 1.5-fold higher, respectively, in Fol-NC-*anti*miR21 and in Apt-NC-*anti*miR21 treated cells *versus* negative controls.

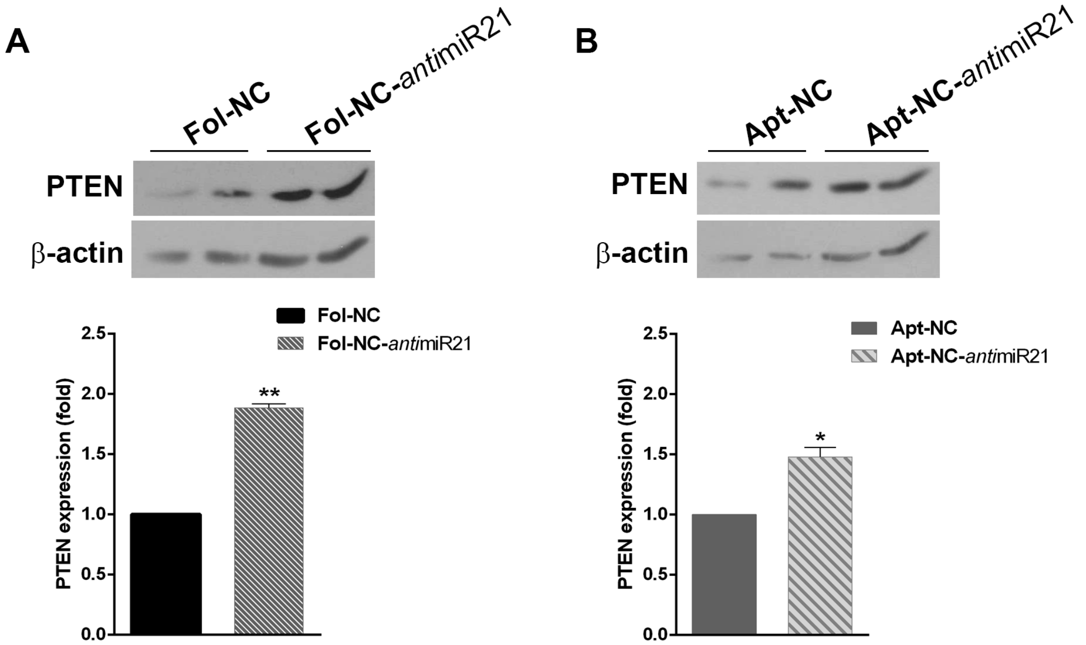

Figure S3 Western blots of PTEN in MDA-MB-231 cell lysates. Histograms on the panels below show the densitometric analysis performed on three different experiments. Values are expressed as mean ± S.E.M. Statistical significance: *P < 0.05 and **P < 0.01 (Student's t-test).

S5. Standard curve of free Dox fluorescence

A calibration curve was obtained by measuring fluorescence (Excitation wavelength: 485 nm; Emission wavelength: 590 nm) of free Dox samples at known concentration using a Spark® Multimode Microplate Reader (TECAN).

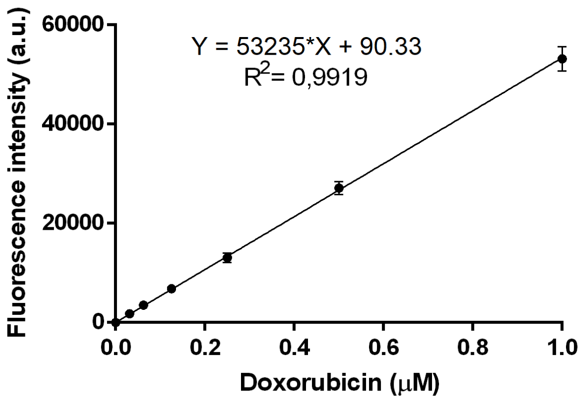

Figure S4 Standard curve of free Dox.

In the experiment shown in Figure 3 of the manuscript the concentration of intercalated Dox was calculated considering that nanostructures were used at 11 µM bp, and the intercalation mix Dox:base-pairs (bp) is at 1:2 ratio. Since initial Dox in the mix is 5.5 µM and the calculated % of intercalated Dox is 22.5% the calculated intercalated Dox is 1.2 µM.

S6. Time and dose-dependent cytotoxic effect of free Dox on HeLa and MDA-MB-231 cells

MDA-MB-231 and HeLa cells were treated with increasing concentrations of Doxorubicin ranging from 0 to 10 µM for 24 (Figure S6A) and 48 h (Figure S6B). MDA-MB-231 cells display a reduced sensitivity to Dox, reaching a plateau even when treated with the highest concentrations.

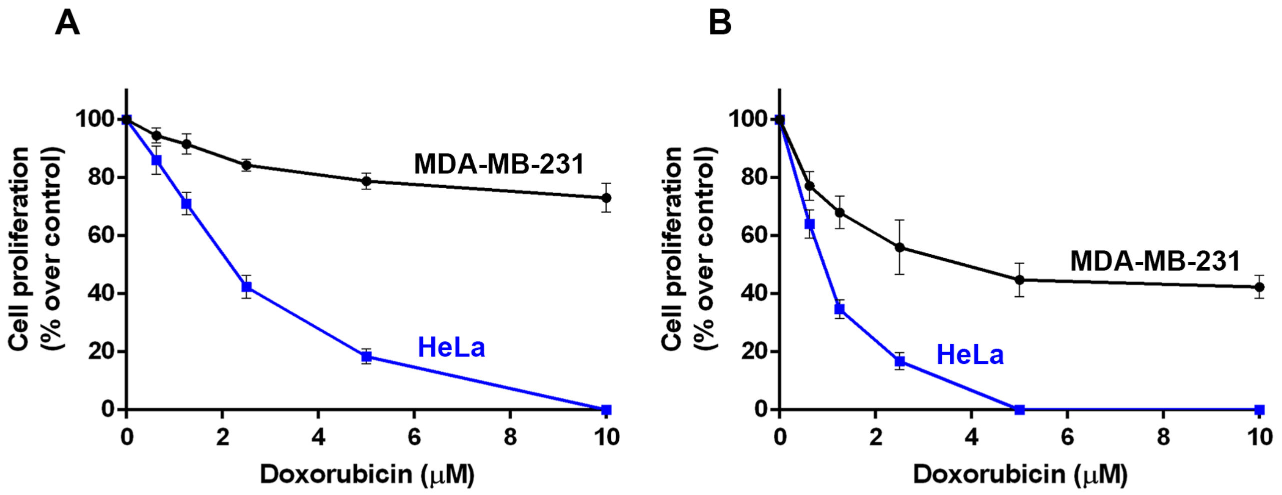

Figure S5 MTS assay of HeLa and MDA-MB-231 cells treated with increasing concentration of free Doxorubicin for 24 h (A) and 48 hours (B). The data represent mean ± S.E.M. of three separate experiments. The values are the mean of six replicates, normalized on cell proliferation of untreated cells.

S7. Confocal analysis of Dox-loaded Fol-Apt-NC

MDA-MB-231 cells were seeded onto poly-L-lysine-coated glass cover-slides at a density of 3 x 10^5 cells/35mm culture dish. Confocal microscopy experiments were performed by incubating cells with either 1,6 μM free Dox or 60nM Dox-loaded Fol-Apt-NC for 2 h at 37 °C, fixed with 4% paraformaldehyde, and permeabilized with Tris/Triton (Tris HCl 0.1 M, Triton 0.1%, pH 7.6). Confocal fluorescence microscopy images were obtained with a Nikon A1R+ laser scanning confocal microscope (Nikon Instruments, Tokyo, Japan) at 60x magnification.

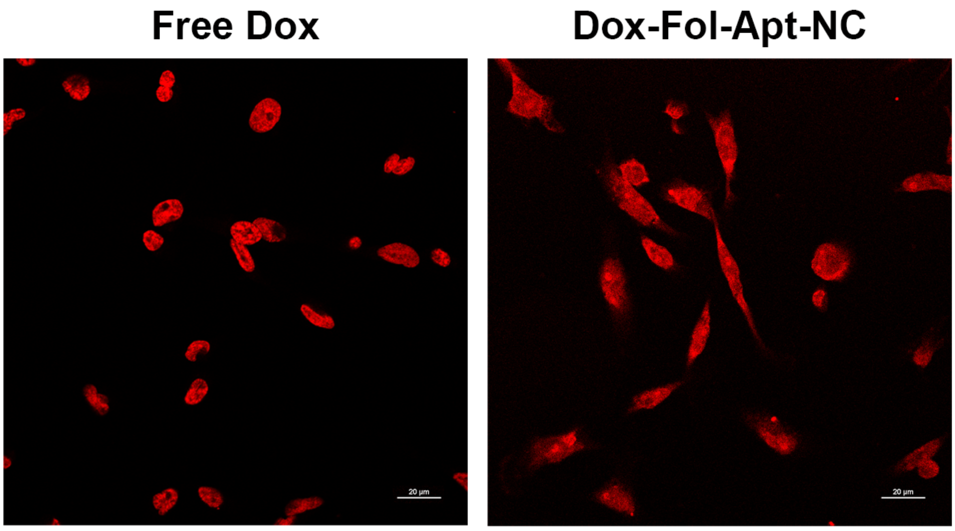

Figure S6 Intracellular distribution of Dox. Confocal images of MDA-MB-231 cells treated with free Dox (left panel) or Dox-loaded Fol-Apt-NC (right panel) for 2h. Scale bar: 20 μm.
